# A literature-based similarity metric for biological processes

**DOI:** 10.1186/1471-2105-7-363

**Published:** 2006-07-26

**Authors:** Monica Chagoyen, Pedro Carmona-Saez, Concha Gil, Jose M Carazo, Alberto Pascual-Montano

**Affiliations:** 1 Biocomputing Unit. Centro Nacional de Biotecnologia – CSIC, Madrid, Spain; 2 Dpto. Arquitectura de Computadores y Automatica. Universidad Complutense de Madrid, Madrid, Spain; 3 Dpto. Microbiologia II. Facultad de Farmacia. Universidad Complutense de Madrid, Madrid, Spain; 4 Unidad de Proteomica UCM – Parque Cientifico de Madrid, Madrid, Spain

## Abstract

**Background:**

Recent analyses in systems biology pursue the discovery of functional modules within the cell. Recognition of such modules requires the integrative analysis of genome-wide experimental data together with available functional schemes. In this line, methods to bridge the gap between the abstract definitions of cellular processes in current schemes and the interlinked nature of biological networks are required.

**Results:**

This work explores the use of the scientific literature to establish potential relationships among cellular processes. To this end we haveused a document based similarity method to compute pair-wise similarities of the biological processes described in the Gene Ontology (GO). The method has been applied to the biological processes annotated for the *Saccharomyces cerevisiae *genome. We compared our results with similarities obtained with two ontology-based metrics, as well as with gene product annotation relationships. We show that the literature-based metric conserves most direct ontological relationships, while reveals biologically sounded similarities that are not obtained using ontology-based metrics and/or genome annotation.

**Conclusion:**

The scientific literature is a valuable source of information from which to compute similarities among biological processes. The associations discovered by literature analysis are a valuable complement to those encoded in existing functional schemes, and those that arise by genome annotation. These similarities can be used to conveniently map the interlinked structure of cellular processes in a particular organism.

## Background

The post-genomic era is driving molecular cell biology in a science that, in addition to the assignment of functions to individual proteins or genes, is now trying to cope with the complex sets of molecules that interact to perform cellular functions [1]. The different aspects of these cellular functions might be described in terms of a multi-scale 'biological atlas' [2]. However, the construction of such atlas is not straightforward, as there is a need to integrate and relate the information obtained from genome-wide experimental data with already existing functional schemes [3].

The construction of functional schemes typically starts from a conceptualization of the repertoire of cellular and molecular functions, and use these to describe the roles of individual gene products. A very illustrative example is the Gene Ontology (GO) project [4], a collaborative effort to address the need for consistent descriptions of gene products in different databases. The GO collaborators are developing three structured, controlled vocabularies (ontologies) that describe gene products in terms of their associated biological processes, cellular components and molecular functions in a species-independent manner. In addition to the development and maintenance of the ontologies themselves, they produce associations between the ontologies and the genes and gene products in the collaborating databases.

Meanwhile genome-wide analytical methodologies are generating large amounts of data related to genes and proteins at different functional levels. For example, recent computational and experimental research provides evidence that functional modules are indeed basic functional units of cellular processes [5-8]. In order to assist in the interpretation of genome-wide data, diverse computational methods that analyze functional information have been developed. One of the most accepted approaches is the enrichment assessment of functional annotations in a gene list (for a review see [9]). The aim of this approach is to discover the biological processes that are statistically relevant in an experimental dataset, i.e. the processes that are characteristic of a particular biological system in a particular state.

In addition to this ontological analysis, there are a number of methods that compare gene/proteins based on their functions. A first group relies on the analysis of functional annotations: establishing a distance or similarity metric using the ontology structure [10-12] or comparing gene vector-based representations derived from a GO association matrix [13]. Ontology-based metrics are increasingly used in quite different bioinformatics applications (e.g. validation [14] and prediction [15] of protein-protein interactions, priorization of disease candidate genes [16], missing value estimation in microarray data [17]). A second group comprises several literature analysis approaches. Among them, a number of methods use different document similarity measurements to establish potential gene relationships and to perform functional classification [18-22].

While much research has been devoted to the development of literature analysis methods to compare functional information at the gene and protein level, little research has been done on the analysis and comparison of the biological processes themselves. On the other hand, several functional scheme relationships have been studied through the analysis of experimental data (e.g. protein interaction [23], genetic interaction [24] and gene expression data [25]), as well as genome annotation [26,27] and linguistic content [28], but not text analysis.

This work explores the use of the scientific literature to establish relationships among biological processes in the context of a single organism. To this end, we define a similarity score between biological processes using Latent Semantic Analysis [29] of relevant documents. To verify that our proposal is valid, we created a pair-wise similarity matrix of the GO biological process annotated for *Saccharomyces cerevisiae *in the *Saccharomyces *Genome Database (SGD) [30], and compared our results with those obtained using two previously reported ontology-based measurements [11,12]. GO annotations in SGD provide the repertoire of biological processes of a particular organism, *S. cerevisiae*, as well as the references to relevant publications supporting the assignments of GO terms to gene products. Finally, we demonstrate the value of such analysis by investigating revealed (dis/)similarities that cannot be obtained using ontology-based metrics. These findings also highlight some of the limitations of currently used graph-based methods.

## Results

GO defines a biological process as a series of events accomplished by one or more ordered assemblies of molecular functions. In our work, this definition is instantiated as a set of bibliographic references related to a particular GO biological process term. To ensure that the documents analyzed contain relevant information, the literature set was constructed from the bibliographic evidences supporting associations of GO biological process categories to gene products in a particular organism.

We applied our method to establish the functional similarity of GO Biological Process annotations for *S. cerevisiae *as provided in the SGD [30]. From the more than 10,500 biological process categories in GO, 1147 terms were annotated in the SGD association file.

Bibliographic references are provided in GO as evidence of a particular gene product being involved in a particular biological process. We compiled all the bibliographic references for a GO biological process, independently of the gene product annotated and independently of the GO hierarchy. Therefore, in this work the literature of a biological process comprises just the references directly associated in the SGD GO annotation file. No ontological relationships among biological processes were considered in the compilation of references in order to objectively compare our approach with ontology-based metrics.

From the 1147 biological process categories found in the SGD annotation file, 1132 contained at least one reference in PubMed [31] included as evidence. This resulted in a list of 1132 GO biological process categories (see project web page [32]). A final total of 3,814 distinct articles were analyzed with an average of 4.5 references per process (the maximum number 76, corresponding to GO:0006468 'protein amino acid phosphorylation').

Using this list, we proceeded to construct a term-frequency vector representation from the literature associated with each biological process. The resulting term-process matrix after this procedure contains 1132 vectors (processes) and 12,409 variables (terms).

SVD factorization with 200 factors, which were selected by the scree test, was applied to this term-process frequency matrix. This factorization rank accounts for the 50.7 % of the variance of the original data. SVD provided a reduced dimensionality space in which relationships among biological processes could be robustly established. The similarity between any two biological processes (slit) was calculated as the cosine of the angle between the process vectors as represented in **VS **matrix (see Methods section).

### Evaluation

Our assumption, which is further justified by our results, is that similarities between biological processes based on the analysis of their associated literature are indeed accurate. That is, biological processes described by similar document representations are certainly related.

In the absence of a gold standard to validate similarities among biological processes, we compared our results with two sources of information: ontological relationships and gene product co-annotation. To this aim, only references directly associated to a given GO term were considered relevant for that particular process. This ensured that conserved relationships were genuinely discovered from document similarity.

In order to ensure that a minimum of textual information was analyzed for each biological process, the comparison was performed using the subset of 282 GO biological processes that contained at least 5 bibliographic references (see project web page [32]). Nevertheless, the latent semantic space was constructed using all the biological processes annotated for *S. cereviae*.

We compared our results with those obtained using two previously reported ontology-based metrics:

• **Lin similarity (slin)**: We have computed the semantic similarity between biological processes using the information content-based definition proposed by Lin, 1998 [33] and used in [11] to compare proteins. This measure makes explicit use of the ontological structure, as well as the frequency of annotation (it will therefore different when computed from different sets of gene product annotations). In [10,11], authors were mostly interested in the semantic similarity between proteins, rather than GO terms *per se*. The similarity between two proteins was established as the average similarity between all annotated terms. In our work, the similarity is simply applied to the GO biological process terms.

• **Czekanowski-Dice similarity (scd)**: This similarity is based on Czekanowski-Dice formula as used in the GO-Proxy tool described in [12]. In GO-Proxy, the formula is applied to calculate the similarity between genes, using all their GO annotations. Rather than genes, we apply the Czekanowski-Dice distance formula to compute the similarity between GO biological processes.

In addition, a measure of similarity based on gene product annotation was also used for comparison purposes. This measure was computed as a modified version of [26], where each GO term is first represented as a vector of gene products. This representation is built from the GO annotations corresponding to a given organism (*S. cerevisiae *in our case). Details are provided in the Methods section.

We compared the similarities computed for all pairs of the 282 processes in the validation subset using our literature analysis to those obtained by the three metrics above. Histograms corresponding to the pair-wise similarities of this set using the four metrics are included in the supplementary material (Additional file 1).

Figure 2 provides three boxplots, one for each measure used as evaluation, where similarity values are plotted for groups of process pairs categorized by their literature-based similarity. These results show that there is an overall agreement between the similarity computed from the literature and the two ontology-based similarities since increasing values in literature-based similarity correspond on average to increasing values on both ontology-based similarity metrics. An analogous overall agreement is observed with the similarity computed from gene annotation. We also noticed that process pairs with high literature similarities show more comparable results according to Czekanowski-Dice similarity (Figure 2b) than to Lin similarity (Figure 2a). A boxplot comparing both ontology-based similarities is provided in the supplementary material (Additional file 2).

To test whether the similarities obtained by our analysis was due to document semantic similarity and not just gene co-annotation we also analysed the relationship of similarity values along the number of common genes and common references for all process pairs in the evaluation subset. Additional file 3 contains plots of the literature-based similarity against (a) the number of genes shared by any two biological processes (no more than 3 genes are shared by any two processes); (b) the normalised number of references shared by any two biological processes. As expected, literature-based similarity increases as the number of common references increases, although high similarities can also be found even if there are no shared references between processes.

However, biological process pairs within each literature-based similarity interval have ontology-based similarity values expanding a wide range. Additional file 4 contains a table with correlation coefficients computed for the four similarity metrics. Correlation with ontology-based metrics increases significantly when only inclusion relationships are analysed (i.e. those process pairs in which one process contains all the genes associated with the other). This is an indicator that, although the literature-based similarity reveals close ontological relationships, in most of the cases the literature and the ontology contain different information from which to establish associations. In order to discover the nature of such information we analyzed those biological process pairs for which we obtained most contradictory values, providing the results in the following sections.

### New similarities revealed

In an attempt to discover new insights from the similarity metric we are proposing, we wanted to check whether our literature analysis is able to find relevant similarities among biological processes which are not revealed by metrics based on ontological relationships. To this aim, we selected pairs of processes that fulfil the following criteria:

• They are very similar according to literature-based metric, being among the 1% of pairs with highest similarity (slit > 0.4345).

• They have similarities less than average according to both ontology-based metrics (scd < 0.4025 and slin < 0.14).

The selection consists of 49 pairs of biological processes (see Additional file 5), where 30 pairs (61.22%) have at least 1 gene in common (annotated with both processes), and 19 (38.78%) have no common genes (genes shared by two processes are obtained among the genes annotated for a GO term or any of their descendants).

Here, we take a closer look at the ten most similar processes in the selection (see Table 1). Most of the process pairs in this top 10 list either have no common genes or one gene co-annotated. Among the first, there are several metal ion transport and corresponding homeostasis processes (namely 'high affinity iron ion transport' and 'iron ion homeostasis', 'copper ion import'/'intracellular copper ion transport' and 'copper ion homeostasis'). Although these processes are distant attending to the GO hierarchy their associated literature reveals that there is a potential relationship among them. Indeed, GO defines metal ion homeostasis processes as the regulation of the levels, transport, and metabolism of metal ions within a cell or between a cell and its external environment. Therefore, a certain level of relationship between homeostasis and transport mechanisms is expected by definition.

Another pair of biological processes with high similarity according to our analysis is 'cell wall chitin biosynthesis' and 'cell budding'. It is known that *S. cerevisiae *reproduces asexually by budding. Immediately prior to and during cellular division chitin, a minor component of the cell wall, is produced and localizes predominantly at the site of bud emergence [34]. Therefore, it is reasonable to find a high similarity score between 'cell wall chitin biosynthesis' and 'cell budding'. In contrast, the ontology just highlights the relationships of 'cell budding' to division, growth and reproduction, while relates 'cell wall chitin biosynthesis' to metabolism and cell organization and biogenesis.

Other similar pairs can be explained by the analysis of a common gene. This is the case of the 'intracellular copper ion transport' and the 'cytochrome c oxidase complex assembly' processes, which are co-associated to *COX17*. Cytochrome c oxidase assembly is dependent on the insertion of different types of cofactors, including three copper ions [35]. Cox17p is involved in copper ion trafficking to the mitochondrion and plays an essential role in the assembly of the cytochrome c-oxidase [36].

'DNA replication checkpoint' is similar to two processes related to DNA replication: 'DNA strand elongation' and 'DNA replication initiation'. GO defines 'DNA replication checkpoint' as a signal transduction based surveillance mechanism that prevents the initiation of mitosis until DNA replication is complete, thereby ensuring that progeny inherit a full complement of the genome. However, GO includes 'DNA replication checkpoint' as a child process of 'regulation of cell cycle', while it does not provide direct relationship to DNA replication processes.

One pair, 'phosphoinositide dephosphorylation' and 'inositol lipid-mediated signaling', has no direct hierarchical relationship in GO up to the 'cellular process' category. However, in SGD association file the seven genes that are annotated as 'phosphoinositide dephosphorylation' are also annotated as 'inositol lipid-mediated signaling'. These genes are: *INP51, INP52*, *INP53*, *INP54*, *SAC1*, *TEP1*, and *YMR1*. This degree of overlapping is also reflected at the bibliographic references, as the five references attached to 'phosphoinositide dephosphorylation' in SGD are a subset of the ten evidences attached to 'inositol lipid-mediated signaling'. Similarly, 13 of the 17 genes that are annotated as 'Rho protein signal transduction' in SGD are also annotated as 'establishment of cell polarity (sensu Fungi)' (namely, *BEM4*, *BNI1*, *BOI1*, *BOI2*, *BUD6*, *CDC42*, *CLA4*, *GIC1*, *GIC2*, *PEA2*, *SLG1*, *SPA2*, and *SPH1*). Among the 19 references analyzed for 'establishment of cell polarity (sensu Fungi)', we found 5 of the 8 references attached to 'Rho protein signal transduction'. In these two cases, a high similarity between process-documents was therefore expected, as both processes shared a significant number of references.

Finally, the 'mitochondrial signaling pathway' is related, according to the literature metric, to 'protein localization'. GO defines the 'mitochondrial signaling pathway' as a series of molecular signals that forms a pathway of communication from the mitochondria to the nucleus and initiates cellular changes in response to changes in mitochondrial function. In SGD, this pathway contains 6 genes (*LST8*, *MKS1*, *RTG1*, *RTG2*, *RTG3*, and *TOR1*). In contrast, 'protein localization' is a broad category (e.g. it comprises 'protein transport' processes) that includes, as GO defines it, the processes by which a protein is transported to, or maintained in, a specific location. Among all genes belonging to this category, only 5 are directly associated in the SGD GO association file (*PUF4*, *RTG1*, *SHR5*, *SNX3*, and *VTC2*). As such, this is a very heterogeneous group of genes and thus the bibliographic references included in our analysis. The similarity is explained by *RTG1*, a gene encoding a transcription factor co-annotated with both categories. Mitochondria-to-nuclear signaling is regulated by the subcellular localization of Rtg1p and Rtg3p [37].

### Hidden similarities

In addition to the previous study, we also wanted to check the opposite effect: what kind of ontology-based significant similarities were not contemplated using our literature analysis. To this end, we selected pairs of processes that fulfil the following criteria:

• They are very similar according to both ontology-based metrics, being among the 1% of pairs with highest similarity (scd > 0.875 and slin > 0.7629).

• They have similarities less than average according to the literature (slit < 0.0726).

These resulted in 3 pairs of biological processes (see Table 2). Among the three, only one pair was found to be co-annotated in one gene in the SGD. In this section we explore the reasons why those pairs are highly related in the ontology but not in the literature. In addition, we research what biological processes are similar to each process in the pair according to our literature analysis.

The first pair, 'Rho protein signal transduction' and 'Ras protein signal transduction' are highly similar according to both ontology-based metrics as they are both direct descendants of 'small GTPase mediated signal transduction' category. GO defines this category of signal transduction processes as any series of molecular signals in which a small monomeric GTPase relays one or more of the signals. Hence, the difference between categories lies in the family of proteins that mediates the signaling process (either Ras family or Rho family).

Nevertheless, the cellular processes in which these signalling cascades are involved might be different, and the scientific literature should contemplate them. Most similar processes to 'Rho protein signal transduction' in the whole set of 1132 GO process categories, according to the literature are: 'maintenance of cell polarity (sensu Fungi)' (slit = 0.88), 'small GTPase mediated signal transduction' (slit = 0.86), 'budding cell isotropic bud growth'(slit = 0.85), 'budding cell apical bud growth'(slit = 0.84), and 'establishment of cell polarity (sensu Fungi)' (slit = 0.82). These results are consistent with the roles of the known Rho GTPases in *S. cerevisiae *which are related to cell polarity establishment and maintenance. [38]. In addition, we have searched the significant GO processes of the 17 genes annotated as 'Rho signal transduction' using SGD GO Term Finder tool [39]. Table 3 shows these processes using this gene list. Therefore, our analysis was able to associate 'Rho protein signal transduction' to its direct upper category in GO, as well as to other related processes.

Correspondingly, most similar processes to 'Ras protein signal transduction' according to the literature are: 'G-protein signaling, coupled to cAMP nucleotide second messenger' (slit = 0.98), 'G-protein signaling, adenylate cyclase activating pathway' (slit = 0.96), 'adenylate cyclase activation' (slit = 0.96). Table 4 shows the most significant GO processes annotated to the 17 genes associated with 'Ras protein signal transduction'. Among these genes we find *RAS1 *and *RAS2*, the two *S. cerevisiae *genes encoding for Ras proteins. Ras proteins maintain an essential basal level of cyclic AMP (cAMP) through their activation of adenylate cyclase (Cyr1p) [40]. In *S. cerevisiae*, cAMP activates the cAMP dependent protein kinase A (PKA) (Tpk1p, Tpk2p). All these data agree with the three most similar processes according to the literature.

The second pair, 'signal peptide processing' and 'peptide pheromone maturation' is very similar according to both ontology-based metrics. The reason is that both processes are directly included in the 'protein processing' category, defined in GO as the post translational modification of a protein, particularly secretory proteins and proteins targeted for membranes or specific cellular locations.

Even if both processes involve the modification of proteins, they are related to quite different cellular processes. In this sense, our literature analysis reveals 'cotranslational protein targeting to membrane' (0.70 similarity), as the most similar process to 'signal peptide processing'. In this case, GO offers no definition for 'signal peptide processing' category. We therefore analyzed the 7 genes associated to this term in the SGD. A subset among this group contains 4 subunits of the signal peptidase complex which cleaves the signal sequence of proteins targeted to the endoplasmic reticulum (*SEC11*, *SPC1*, *SPC2*, *SPC3*), which justifies the similarity to 'cotranslational protein targeting to membrane'.

In the same way we analysed most similar processes obtained for 'peptide pheromone maturation' by literature analysis: 'protein amino acid farnesylation' (slit = 0.91) and 'negative regulation of transcription from RNA polymerase II promoter by pheromones' (slit = 0.61). *S. cerevisiae *uses two peptide pheromones, known as a-factor and α-factor, for intercellular signalling before mating. Both a-factor and α-factor pheromones are synthesized as larger precursors whose maturation and secretion require rather different posttranslational processing steps and different routes from the ribosome to the exterior of the cell [41]. Accordingly, SGD annotations revealed two groups of genes; those involved in α-factor processing (*KEX2*, *STE13*) and in a-factor processing (*AXL1*, *RAM2*, *RCE1*, *STE14*, *STE23*, *STE24*). Our analysis revealed a relationship to the first modification in the maturation process of the largest group of genes (those involved in a-factor processing). This modification is the coupling of a prenyl lipid, farnesyl, to a cysteine residue four amino acids from the carboxyl terminus, a process which is dependent on the presence of a carboxyl-terminal CAAX motif [41]. In addition to farnesylation, the literature analysis also highlights the similarity to the process involving pheromones that regulates negatively the transcription from an RNA polymerase II promoter.

Finally, the third pair 'calcium ion homeostasis' and 'iron ion homeostasis' are highly similar according to the ontology, as they are both 'metal ion homeostasis' processes. Most similar processes to 'calcium ion homeostasis' are: 'calcium ion transport' (0.98 similarity), 'calcium-mediated signaling' (0.71). Correspondingly, most similar processes to 'iron ion homeostasis' are: 'metal ion homeostasis' (0.95 similarity) to which it is related by hierarchy in the ontology, 'high affinity iron ion transport' (0.94), 'iron ion transport' (0.94), 'siderophore transport' (0.86) and 'mitochondrial iron ion transport' (0.83), corresponding to several ion transport processes.

## Discussion

Cellular processes are accomplished by the cooperative work of diverse molecular entities. Biological processes are currently represented for computational analysis purposes in terms of different, while complementary, information. A biological process can be:

• An enumeration of molecular entities (e.g. the set of gene products of an organism annotated with a particular functional category), together with their corresponding experimental measurements (e.g. expression data);

• An interaction network, a pathway, or a subgraph of a larger network.

• A brief textual description and a set of ontological relationships (e.g. the GO which provides both a graph and definition).

All these types of information should be carefully taken into account in order to get a better understanding of the complex structure of the cellular processes.

This work explores the use of the scientific literature as an additional data representation to describe biological processes. From this data, we established a similarity score that allows the comparison of biological processes. In order to demonstrate the validity of such approach, we computed the pair-wise similarities of the GO biological processes for *S. cerevisiae *as annotated in the SGD database. GO annotations provide the repertoire of biological processes of a particular organism, as well as the references to relevant publications supporting the GO assignments to gene products.

We have compared our results with previously reported ontology-based and gene product annotation similarity measures. Similarities obtained from the literature show an overall agreement with those computed by two previously reported metrics based on ontological relationships [11,12], with a higher correlation in the case of parent/child related processes. They are, therefore, in general agreement with the knowledge on biological processes encoded in the Gene Ontology. Nevertheless, agreement is not significant for those biological processes with no hierarchical relationships in the ontology. In order to provide some hints on the discrepancies obtained by literature and ontology-based methods, we examined those cases where we got most dissimilar results.

We first studied the relationships obtained that were not encoded in the ontology. A possible explanation of the new found relationships is that the literature contains details on the biological processes in a particular context (e.g. a given organism). In contrast, the Gene Ontology is created to assign gene product functional descriptions, and describes biological processes in a species-independent manner. Furthermore, GO relationships are constrained by the 'true path rule', which states that "the pathway from a child term all the way up to its top-level parent(s) must always be true". The ontology it is not developed for describing the knowledge about biological processes, but for gene product annotation. In this sense, any metric just based on the relationships encoded in the ontology will miss the associations between biological processes that arise by genome annotation. In contrast, in our method similarities were computed from a literature collection created in the context of a particular organism. This allowed the establishment of similarities even if the gene products were not co-annotated (e.g. ion transport and ion homeostasis).

Finally, the analysis of processes with high GO-based similarity and low literature-based similarity pointed out some relationships in the GO that might not be relevant in the analysis of the interlinked structure of cellular processes. This is the case of the different protein modification processes, or the relationships among some signalling pathways (e.g. Rho and Ras protein signal transduction processes).

In spite of the relevancy of the reported results, our approach is limited by the availability of a minimum number of relevant references per biological process. Even in the case of a model organism like *S. cerevisiae *for which much functional information is accumulated, there are some biological processes containing just a few relevant references to the scientific literature. Therefore, the differences in the amount of documents associated to each process might bias the similarity measure proposed in this work. To cope with this problem we have used all references related to each process to compute the latent semantic space representation, but the evaluation was only performed on a subset of processes containing a minimum amount of documents. Further research in this topic is desirable.

It is also important to note that the GO annotation guide advises to annotate gene products in each species database to the most detailed level in the ontology that correctly describes the biology of the gene product. Therefore, the annotation of a gene product with a GO term that has descendant terms in the ontology generally implies that the information available does not allow associating a more specific category. This means that some biological processes (especially those upper in the GO hierarchy) might contain very general and quite heterogeneous references (like the case of 'protein localization' reported in the Results section). These sets of articles are therefore not adequate for describing general or abstract process categories. However, we can anticipate that with the methodology presented here, more relevant and sound similarities might be extracted when richer collections of references are compiled.

Correspondingly ontology-based metrics present also some additional limitations beyond those related to the quality and completeness of the relationships established in the ontology. First, biological processes that are not present in the ontology cannot be analysed (e.g. a-factor processing and α-factor maturation processes cannot be compared as they are included as narrow synonyms of the same GO term: peptide pheromone maturation). Second, biological processes described in two different ontologies cannot be compared, unless a mapping is established between the two. In contrast, literature-based similarities can be constructed as long as there are a number of documents describing that particular process, allowing the comparison of processes belonging to different ontologies.

Document similarity approaches are therefore useful, not only for the functional comparison of genes and proteins, but for the discovery of relationships among cellular processes. The high similarity scores obtained for some process pairs reveal that, in terms of the document representation used, some processes are hard to distinguish. Therefore, the literature similarity metric is also valuable for those involved in the development of functional annotation methods relying on related text representations.

## Conclusion

Among the general principles that govern cellular functions, modularity is a characteristic often associated with biological networks. A functional module is described as a discrete entity, composed of several elementary components, with a specialized function that is separable from those of other modules. The higher-level activities of cells might be described by the pattern of connections among their functional modules [1,42,43]. Defined by highly connected regions in interaction networks, functional modules are not fixed entities but can be defined according to different criteria [3]. Metrics establishing similarities among functional annotations are therefore useful in the analysis, validation and interpretation of interaction data, as well as in the computational study of any other genome-wide information.

In this work we demonstrated that the biomedical literature is a valuable source of information from which to obtain potential relationships among cellular processes. In addition to the hierarchical structure of the GO biological process ontology, a pair-wise similarity matrix obtained from the scientific literature can map naturally the interlinked structure of biological processes. Literature-based similarity metrics can therefore complement the ontological relationships established for biological processes, as well as those that arise by gene product annotation. Our results also indicate that a full exploitation of the complementary nature of currently available similarity metrics among biological processes might provide new biological insights (with applications in the validation of experimental data, prediction of functional information and functional annotation from scientific texts), constituting an interesting line for further research. The methodology used in this work is very general, and could be also applied to the comparison of different aspects of biological function, complementing current approaches that rely on genome annotation [27].

## Methods

Briefly, our method proceeds as follows: a broad process-document is constructed for each biological process by concatenating its relevant bibliographic references (abstracts and titles). A vector space representation, namely, a weighted term-frequency vector, is built for each process-document. This term-process matrix (**A**) is mapped by means of a factorization technique, Singular Value Decomposition (SVD), to a lower-dimensional representation. Biological process similarities are computed in the new reduced space obtained in the factorization step. SVD provides a reduced dimensionality space in which relationships among biological processes could be robustly established since it mitigates synonymy, as the rank lowering is expected to merge the dimensions associated with terms that have similar meanings. At the same time this factorization reduces the effect of polysemy, which results in a robust representation space where document similarities can be effectively determined. A full description of the methodology is described in the following subsections.

### Representing GOP-documents

A literature collection was compiled for each GO biological process found in the annotation of the *S. cerevisiae *genome, produced by SGD [30]. SGD annotation file Date: 01/25/2006; Gene Ontology database (01/27/2006).

A set of literature references was compiled for each biological process in the SGD GO association file, using the literature included as supporting evidences. All references were attached to a particular GO process independently of the gene to which they are associated, and independently of the relationships established in the GO structure.

A new document was then constructed for each biological process in Gene Ontology (GOP) by concatenating the titles and the abstracts of all its relevant bibliographic references. Further processing to obtain a vector space representation of the GOP-documents was performed as in [18]. Stop words were eliminated. Word morphological variants were reduced to their root form using the Porter stemming algorithm [44]. Each GOP-document was further represented by a weighted term vector, using the Inverse Document Frequency (TF*IDF) weighting scheme. Formally, the IDF for the *j*^th ^term is calculated as:

idfj=log⁡(Ttj)     (1)
 MathType@MTEF@5@5@+=feaafiart1ev1aaatCvAUfKttLearuWrP9MDH5MBPbIqV92AaeXatLxBI9gBaebbnrfifHhDYfgasaacH8akY=wiFfYdH8Gipec8Eeeu0xXdbba9frFj0=OqFfea0dXdd9vqai=hGuQ8kuc9pgc9s8qqaq=dirpe0xb9q8qiLsFr0=vr0=vr0dc8meaabaqaciaacaGaaeqabaqabeGadaaakeaacqWGPbqAcqWGKbazcqWGMbGzdaWgaaWcbaGaemOAaOgabeaakiabg2da9iGbcYgaSjabc+gaVjabcEgaNnaabmGabaWaaSaaaeaacqWGubavaeaacqWG0baDdaWgaaWcbaGaemOAaOgabeaaaaaakiaawIcacaGLPaaacaWLjaGaaCzcamaabmGabaGaeGymaedacaGLOaGaayzkaaaaaa@40F3@

where *T *is the total number of GOP-documents, and *t*_*j *_is the number of GOP-documents that contain the term *j*.

Thus the weight assigned to term *j *in document *i *under the TF*IDF scheme is:

*D*_*ij *_= *tf*_*ij*_·*idf*_*j *_      (2)

It is important to note that similarities among GOP-documents using this weighting scheme might be biased toward those processes with larger number of references, and those that comprise a more homogeneous set of texts. This is due to the fact that TF*IDF will generally favour frequent terms of processes with numerous references. Alternative weighting schemes (i.e. normalized TF*IDF) or new ways to combine references could be also explored to account for the differences in the amount of relevant articles.

Terms were filtered out if they did not appear in at least 2 of the process-documents. At this stage, a set of *n *GOP-documents is therefore represented as a set of *n *vectors in a *p*-dimensional space of terms, where *p *is typically very high (12,891 in this case).

### Similarity from GOP-documents

Once the GOP-document collection is represented in a vector space model, as an *p *× *n *sparse matrix **A**, the next step was to reduce de dimensionality of such representation. This is accomplished through the application of SVD factorization to find a low-rank approximation (**Â**) to the term-process matrix:

**A ≈ Â = USV**^*t *^      (3)

where **A **is the matrix with *p *terms and *n *processes and **Â **is the rank-*k *matrix with the best possible least-squares-fit to **A**. In this model, **U **is a *p *× *k *matrix with its *p *rows corresponding to terms and its *k *orthogonal columns corresponding to new non-correlated variables. **V**, on the other hand, corresponds to processes that are represented by rows and, like in the case of matrix **U**, its *k *orthogonal columns correspond to the new non-correlated variables. **S **is a *k *× *k *diagonal matrix containing a set of scaling values (known as singular values). SVD factorization has been proven successfully in the clustering of genes and/or proteins by [21], using comparable literature analysis. Alternative methods to perform latent semantic analysis can also be used, e.g. probabilistic latent semantic analysis [45], non-negative matrix factorization (NMF) [46], independent component analysis (ICA) [47], latent Dirichlet allocation (LDA) [48]. These methods can provide added-value to the computation of a similarity score (e.g. factors can be interpreted if only positive weighting is allowed, as in the case of NMF).

**Figure 1:**
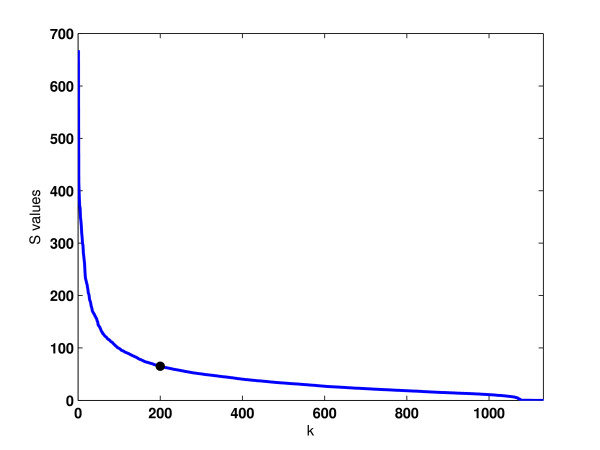
***Singular values***. Singular values corresponding to full-rank SVD factorization (as obtained in **S **matrix) ordered in decreasing magnitude. X axis corresponds to diagonal index. First *k *= 200 largest were kept according to scree test.

**Figure 2:**
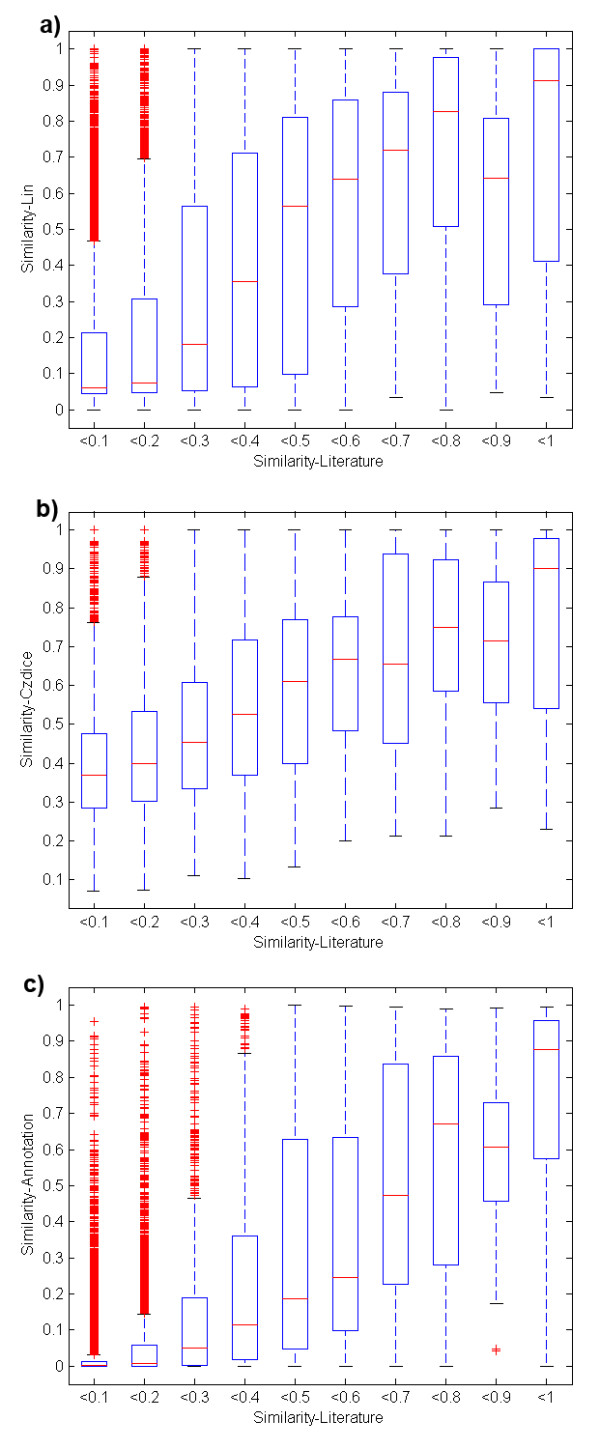
***Comparison of literature similarity with other metrics***. Literature-based similarity boxplot against evaluation metrics. X-axis corresponds to groups of biological process pairs categorized by their literature-based similarity (shown in increasing binned values). Y-axis corresponds to ontology-based similarity computed as a) Lin similarity, b) Czekanowski-Dice similarity, and c) Genome annotation similarity. Boxes expand from lower to upper quartile values, and contain a red line corresponding to median value. Dashed lines extend-ing from each end of the box show the extent of the rest of the data (with a maximum length of 1.5 units of interquartile range). Outliers, data with values beyond the ends of the whiskers, are marked as red crosses.

The amount of dimensionality reduction, that is, the choice of the most important factors is a critical step. It should be large enough to fit the real structure in the data, but small enough such that noise and unimportant details are not considered in the model [49]. In this work we have used the scree test [50] that suggests to perform a full rank factorization and then select the maximum number of factors (*k*) where the smooth decrease of the singular values appears to level off (see figure 1). "Scree" is a term from geology and it represents the rubble at the bottom of a cliff. The idea in the scree test is that if a factor is important, it will have a large variance. Therefore, it is a common practice to order the factors by variance, and plot the variance against the factor number. The scree test recommends keeping the number of factors above the elbow in the plot and thus factor extraction should stop when this line flattens out; in other words, when the consecutive gain in explained variance approaches zero. Using this test a total of 200 factors were selected and used in our analysis.

The dot product between two column vectors of **Â **reflects the extent to which two process-documents have a similar profile of terms. It is easy to prove that this is equivalent to the dot product between rows of the matrix **VS**. Therefore, a similarity metric is obtained for all pairs of biological processes by computing the cosine between each pair of rows of **VS**.

The similarity measure is calculated as:

slit(ci,cj)=cos⁡(θij)=cic′jcic′icjc′j     (4)
 MathType@MTEF@5@5@+=feaafiart1ev1aaatCvAUfKttLearuWrP9MDH5MBPbIqV92AaeXatLxBI9gBaebbnrfifHhDYfgasaacH8akY=wiFfYdH8Gipec8Eeeu0xXdbba9frFj0=OqFfea0dXdd9vqai=hGuQ8kuc9pgc9s8qqaq=dirpe0xb9q8qiLsFr0=vr0=vr0dc8meaabaqaciaacaGaaeqabaqabeGadaaakeaacqWGZbWCcqWGSbaBcqWGPbqAcqWG0baDcqGGOaakcqWGJbWydaWgaaWcbaGaemyAaKgabeaakiabcYcaSiabdogaJnaaBaaaleaacqWGQbGAaeqaaOGaeiykaKIaeyypa0Jagi4yamMaei4Ba8Maei4CamNaeiikaGccciGae8hUde3aaSbaaSqaaiabdMgaPjabdQgaQbqabaGccqGGPaqkcqGH9aqpdaWcaaqaaiabdogaJnaaBaaaleaacqWGPbqAaeqaaOGafm4yamMbauaadaWgaaWcbaGaemOAaOgabeaaaOqaamaakaaabaGaem4yam2aaSbaaSqaaiabdMgaPbqabaGccuWGJbWygaqbamaaBaaaleaacqWGPbqAaeqaaaqabaGcdaGcaaqaaiabdogaJnaaBaaaleaacqWGQbGAaeqaaOGafm4yamMbauaadaWgaaWcbaGaemOAaOgabeaaaeqaaaaakiaaxMaacaWLjaWaaeWaceaacqaI0aanaiaawIcacaGLPaaaaaa@5C88@

where *c*_*i *_corresponds to the i^th ^row in **VS **matrix.

### Other similarity measures

Semantic similarity between GO biological process terms using SGD annotations was calculated as in [11] using Lin metric for a taxonomy [33]. This measure is based on the information content of shared parents of the two ontological terms *c*_*i *_and *c*_*j*_

slin(ci,cj)=2log⁡P(cij)log⁡P(ci)+log⁡P(cj)     (3)
 MathType@MTEF@5@5@+=feaafiart1ev1aaatCvAUfKttLearuWrP9MDH5MBPbIqV92AaeXatLxBI9gBaebbnrfifHhDYfgasaacH8akY=wiFfYdH8Gipec8Eeeu0xXdbba9frFj0=OqFfea0dXdd9vqai=hGuQ8kuc9pgc9s8qqaq=dirpe0xb9q8qiLsFr0=vr0=vr0dc8meaabaqaciaacaGaaeqabaqabeGadaaakeaacqWGZbWCcqWGSbaBcqWGPbqAcqWGUbGBcqGGOaakcqWGJbWydaWgaaWcbaGaemyAaKgabeaakiabcYcaSiabdogaJnaaBaaaleaacqWGQbGAaeqaaOGaeiykaKIaeyypa0ZaaSaaaeaacqaIYaGmcyGGSbaBcqGGVbWBcqGGNbWzcqqGqbaucqGGOaakcqWGJbWydaWgaaWcbaGaemyAaKMaemOAaOgabeaakiabcMcaPaqaaiGbcYgaSjabc+gaVjabcEgaNjabbcfaqjabcIcaOiabdogaJnaaBaaaleaacqWGPbqAaeqaaOGaeiykaKIaey4kaSIagiiBaWMaei4Ba8Maei4zaCMaeeiuaaLaeiikaGIaem4yam2aaSbaaSqaaiabdQgaQbqabaGccqGGPaqkaaGaaCzcaiaaxMaadaqadiqaaiabiodaZaGaayjkaiaawMcaaaaa@6021@

where *c*_*ij *_is the most specific class that subsumes both *c*_*i *_and *c*_*j*_. P(c) is the probability that a object belongs to category *c*, i.e. is the number of gene/gene products associated with *c*, divided by the number of times any GO annotation occurs. Lin similarity is a normalized version of the Resnik similarity [51,52] as used in [10]. Lin formula was chosen as it assures that the similarity between a pair of identical objects is 1, and it is hence defined in the interval [0,1].

The second ontology-based similarity between processes is based on the Czekanowski-Dice formula used by [12] to calculate annotation-based distance between genes. In our work, the distance is applied to calculate the similarity between two biological processes (GO terms) instead to the full set of GO annotations of gene products.

scd(ci,cj)=1#(ciΔcj)#(ci∪cj)+#(ci∩cj)     (4)
 MathType@MTEF@5@5@+=feaafiart1ev1aaatCvAUfKttLearuWrP9MDH5MBPbIqV92AaeXatLxBI9gBaebbnrfifHhDYfgasaacH8akY=wiFfYdH8Gipec8Eeeu0xXdbba9frFj0=OqFfea0dXdd9vqai=hGuQ8kuc9pgc9s8qqaq=dirpe0xb9q8qiLsFr0=vr0=vr0dc8meaabaqaciaacaGaaeqabaqabeGadaaakeaacqWGZbWCcqWGJbWycqWGKbazcqGGOaakcqWGJbWydaWgaaWcbaGaemyAaKgabeaakiabcYcaSiabdogaJnaaBaaaleaacqWGQbGAaeqaaOGaeiykaKIaeyypa0JaeGymaeZaaSaaaeaacqGGJaWicqGGOaakcqWGJbWydaWgaaWcbaGaemyAaKgabeaakiabfs5aejabdogaJnaaBaaaleaacqWGQbGAaeqaaOGaeiykaKcabaGaei4iamIaeiikaGIaem4yam2aaSbaaSqaaiabdMgaPbqabaGccqGHQicYcqWGJbWydaWgaaWcbaGaemOAaOgabeaakiabcMcaPiabgUcaRiabcocaJiabcIcaOiabdogaJnaaBaaaleaacqWGPbqAaeqaaOGaeyykICSaem4yam2aaSbaaSqaaiabdQgaQbqabaGccqGGPaqkaaGaaCzcaiaaxMaadaqadiqaaiabisda0aGaayjkaiaawMcaaaaa@5D26@

where # denotes the number of elements in the set, and Δ is the symmetrical difference between the two sets. This metric emphasizes the importance of shared ancestors in the hierarchy by giving more weight to commonalities than to differences.

Similarity using genome annotation was estimated by a modified version of [26], where a binary annotation matrix of GO process terms by gene products (**G**) is first obtained from SGD and the GO hierarchy (i.e. a gene product is associated with a GO process and all its parents). In this case, GO processes are therefore represented as vectors of gene products. In the same way as in the case of document similarity, we applied SVD factorization to **G **(**G **= **USV**^**t**^) in order to find a low-rank approximation **Ĝ **(*k *= 100 factors selected by scree test). Similarity is computed as the cosine of the angle between process vectors (rows in **VS**) as in equation (4).

## Authors' contributions

MC conceived the work, performed the analyses and drafted the manuscript. PCS and CG assessed and revised critically the results. APM and JMC revised both the methodology and manuscript critically for important intellectual content. All authors participated in writing, revising and approving the final manuscript.

## Supplementary Material

Additional file 1 ***Histograms***. This file contains three histograms corresponding to the pair-wise similarities among biological processes in the 282 subset used for validation, as obtained by literature analysis, the GO ontology structure (using Lin and Czekanowski-Dice formulae) and *S. cerevisiae *genome annotation. Click here for file

Additional file 2 ***Comparison of ontology-based similarities***. This file contains the boxplot of Lin similarity along different intervals of Czekanowski-Dice similarity. Click here for file

Additional file 3 ***Correlation with shared genes/references***. This file contains plots of the literature-based similarity against (a) the number of genes shared by any two biological processes (note than no more than 3 genes are shared by any two processes); (b) the normalised number references shared by any two biological processes. Click here for file

Additional file 4 ***Correlation among similarity metrics***. This file contains the correlation coefficients (using Pearson, Spearman, Kendall and uncentered dot product methods) among the four similarity metrics used for the evaluation set. Click here for file

Additional file 5 ***Similar biological processes according to the literature***. This file contains the 49 biological process pairs which are similar according to literature and similar less than average according to both ontology-based metrics. The first 10 pairs correspond to Table 1 in the full text article. Click here for file

## Figures and Tables

**Table 1:** *Similar biological processes according to the literature*. Top 10 biological process pairs most similar according to the literature while dissimilar (similar less than average) according to the ontology. Slit (literature-based similarity); Scd (Czekanowski-Dice similarity); Slin (Lin similarity); CG (number of genes co-annotated). Total number of genes annotated with each biological process term is shown in brackets.

Slit	Scd	Slin	CG	Biological Process A	Biological Process B
0.94	0.37	0.04	0	high affinity iron ion transport (5)	iron ion homeostasis (29)
0.94	0.23	0.03	7	phosphoinositide dephosphorylation (7)	inositol lipid-mediated signalling (13)
0.91	0.37	0.03	0	copper ion import (5)	copper ion homeostasis (7)
0.82	0.29	0.04	13	Rho protein signal transduction (17)	establishment of cell polarity (sensu Fungi) (104)
0.77	0.36	0.04	1	DNA replication checkpoint (5)	DNA strand elongation (30)
0.76	0.40	0.00	1	mitochondrial signaling pathway (6)	protein localization (287)
0.75	0.21	0.04	0	cell wall chitin biosynthesis (7)	cell budding (81)
0.75	0.32	0.03	1	intracellular copper ion transport (6)	cytochrome c oxidase complex assembly (9)
0.73	0.32	0.03	0	intracellular copper ion transport (6)	copper ion homeostasis (7)
0.72	0.36	0.04	1	DNA replication checkpoint (5)	DNA replication initiation (25)

**Table 2:** *Similar biological processes according to the ontology*. Biological process pairs most similar according to the ontology while dissimilar (similar less than average) according to the literature. Slit (literature-based similarity); Scd (Czekanowski-Dice similarity); Slin (Lin similarity); CG (number of genes co-annotated). Total number of genes annotated with each biological process term is shown in brackets.

Slit	Scd	Slin	CG	Biological process A	Biological Process B
0.06	1	0.82	0	Rho protein signal transduction (17)	Ras protein signal transduction (17)
0.04	0.97	0.80	0	signal peptide processing (7)	peptide pheromone maturation (8)
0.05	0.96	0.78	1	calcium ion homeostasis (7)	iron ion homeostasis (29)

**Table 3:** *'Rho protein signal transduction' related processes by gene co-annotation*. Significant shared GO processes in the 17 'Rho protein signal transduction' genes

**GO Process**	**Frequency**	**Probability**	**Genes**
Rho protein signal transduction	100%	1.77E-45	BEM4, BNI1, BOI1, BOI2, BUD6, CDC42, CLA4, GIC1, GIC2, PEA2, RHO5, SLG1, SPA2, SPH1, TOR2, WSC2, WSC3
morphogenesis	82.3%	3.83E-21	BEM4, BNI1, BOI1, BOI2, BUD6, CDC42, CLA4, GIC1, GIC2, PEA2, SLG1, SPA2, SPH1, TOR2
establishment of cell polarity (sensu Fungi)	76.4%	4.19E-21	BEM4, BNI1, BOI1, BOI2, BUD6, CDC42, CLA4, GIC1, GIC2, PEA2, SLG1, SPA2, SPH1
development	82.3%	1.54E-15	BEM4, BNI1, BOI1, BOI2, BUD6, CDC42, CLA4, GIC1, GIC2, PEA2, SLG1, SPA2, SPH1, TOR2
actin cytoskeleton organization and biogenesis	58.8%	6.80E-15	BEM4, BNI1, BUD6, PEA2, SLG1, SPA2, SPH1, TOR2, WSC2, WSC3
actin filament-based process	58.8%	9.85E-15	BEM4, BNI1, BUD6, PEA2, SLG1, SPA2, SPH1, TOR2, WSC2, WSC3
asexual reproduction	52.9%	5.78E-14	BNI1, BUD6, CDC42, CLA4, GIC1, GIC2, PEA2, SPA2, SPH1
cell budding	52.9%	5.78E-14	BNI1, BUD6, CDC42, CLA4, GIC1, GIC2, PEA2, SPA2, SPH1

**Table 4:** *'Ras protein signal transduction' related processes by gene co-annotation*. Significant shared GO processes in the 17 'Ras protein signal transduction' genes

**GO Process**	**Frequency**	**Probability**	**Genes**
Ras protein signal transduction	100%	1.77E-45	BMH1, BMH2, CDC25, CYR1, ERI1, IRA1, IRA2, MSI1, RAS1, RAS2, RPI1, SDC25, SHR5, SRV2, TPK1, TPK2, YLL017W
pseudohyphal growth	29.4%	8.76E-08	BMH1, BMH2, RAS2, TPK1, TPK2
G-protein signaling, coupled to cAMP nucleotide second messenger	17.6%	1.11E-07	CYR1, RAS1, RAS2
G-protein signaling, adenylate cyclase activating pathway	11.7%	1.02E-05	RAS1, RAS2
development	41.1%	1.93E-05	BMH1, BMH2, CDC25, CYR1, IRA1, MSI1, RAS2
sporulation (sensu Fungi)	23.5%	5.74E-05	BMH1, BMH2, IRA1, RAS2
