# Supplementary material for: A literature-based similarity metric for biological processes
Source: BMC Bioinformatics. 2006 Jul 26;7:363. doi: 10.1186/1471-2105-7-363 (PMC1579237; doi:10.1186/1471-2105-7-363)
Supplement: Additional file 1 — Histograms. This file contains three histograms corresponding to the pair-wise similarities among biological processes in the 282 subset used for validation, as obtained by literature analysis, the GO ontology structure (using Lin and Czekanowski-Dice formulae) and S. cerevisiae genome annotation. [file 1471-2105-7-363-S1.PDF]

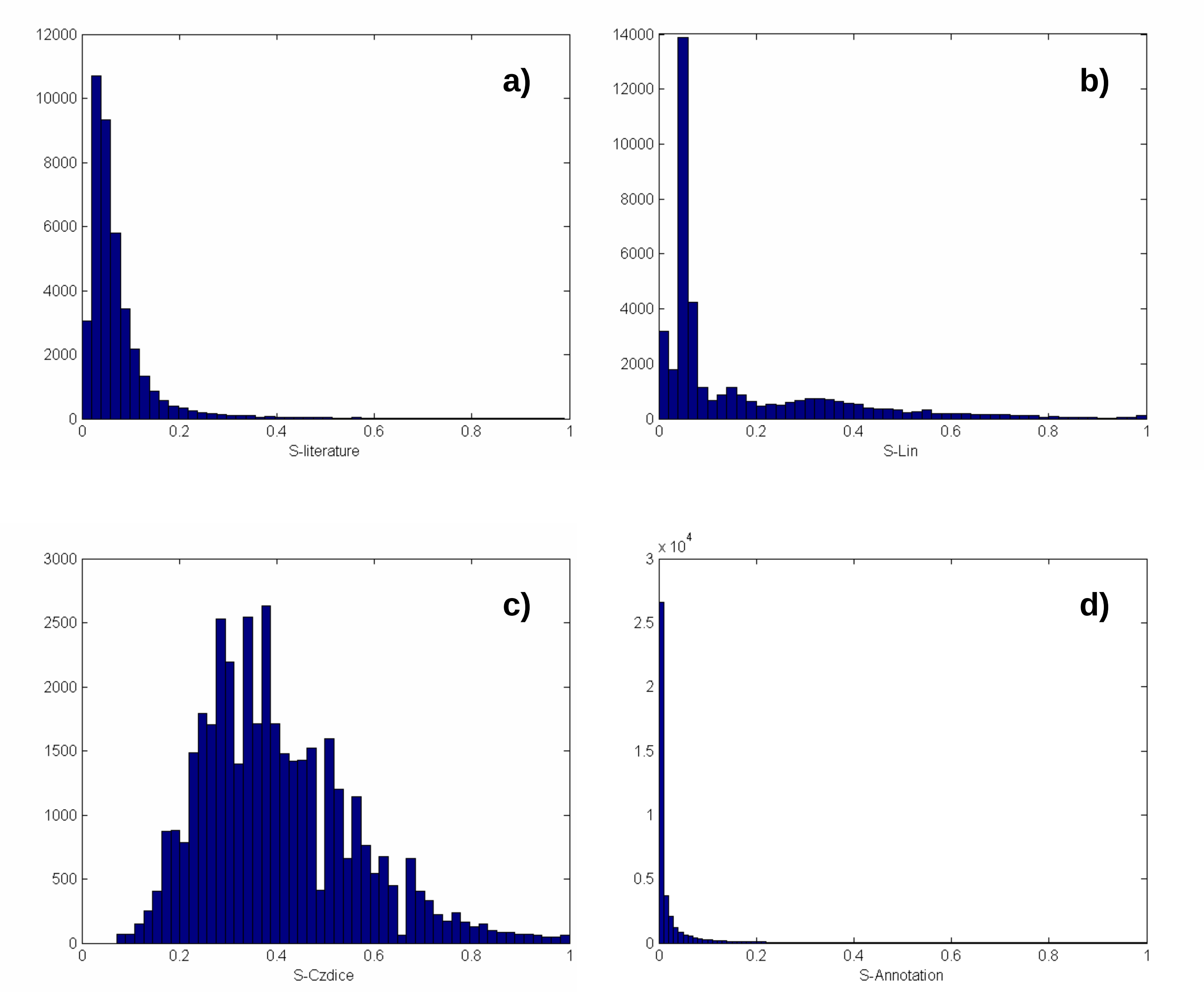

Histograms:Corresponding to the pair-wise similarities obtained for the 282 biological processes used for valudation. a) Literature similarity. b) Ontology-based Lin similarity. c) Ontology-based Czekanowski-Dice similarity. d) Genome annotation similarity
