# Supplementary material for: A literature-based similarity metric for biological processes
Source: BMC Bioinformatics. 2006 Jul 26;7:363. doi: 10.1186/1471-2105-7-363 (PMC1579237; doi:10.1186/1471-2105-7-363)
Supplement: Additional file 2 — Comparison of ontology-based similarities. This file contains the boxplot of Lin similarity along different intervals of Czekanowski-Dice similarity. [file 1471-2105-7-363-S2.PDF]

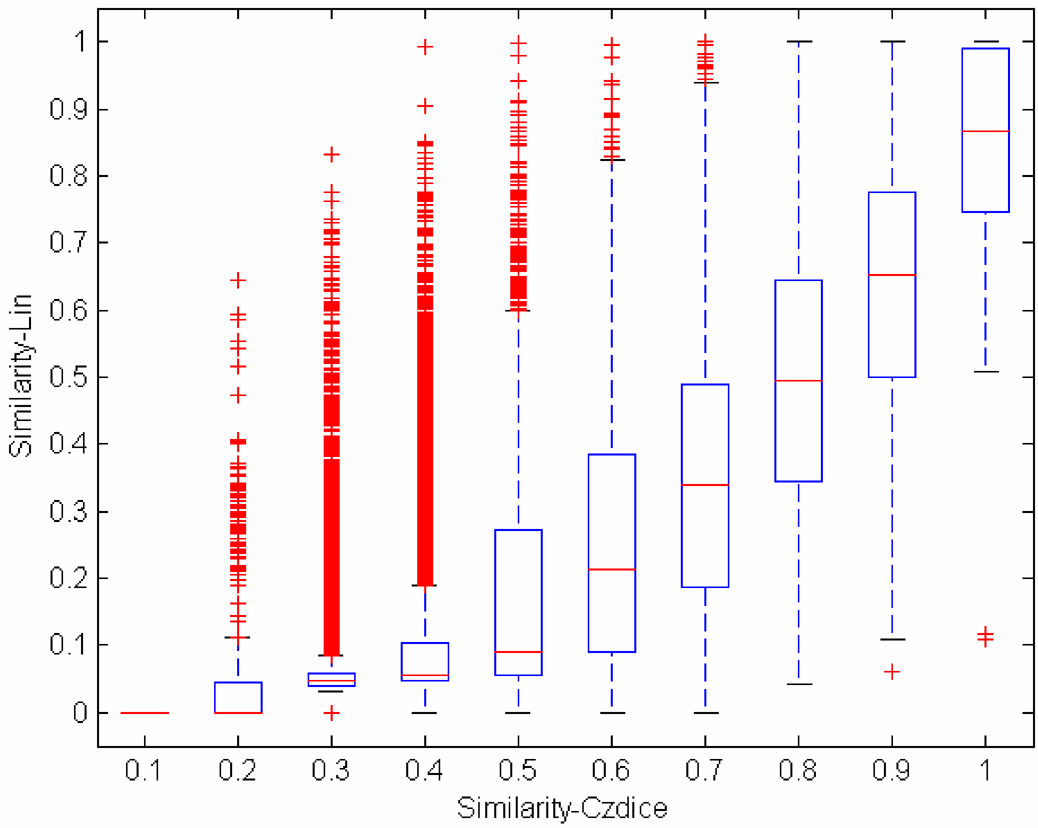

Ontology-based Lin similarity boxplot: X-axis corresponds to groups of biological process pairs categorized by their ontology-based similarity (shown in increasing binned values) computed as Czekanowski-Dice similarity. Y-axis corresponds to Lin similarity. Boxes expand from lower to upper quartile values, and contain a red line corresponding to median value. Dashed lines extending from each end of the box show the extent of the rest of the data (with a maximum length of 1.5 units of interquartile range). Outliers, data with values beyond the ends of the whiskers, are marked as red crosses.
