# Supplementary material for: A literature-based similarity metric for biological processes
Source: BMC Bioinformatics. 2006 Jul 26;7:363. doi: 10.1186/1471-2105-7-363 (PMC1579237; doi:10.1186/1471-2105-7-363)
Supplement: Additional file 3 — Correlation with shared genes/references. This file contains plots of the literature-based similarity against (a) the number of genes shared by any two biological processes (note than no more than 3 genes are shared by any two processes); (b) the normalised number references shared by any two biological processes. [file 1471-2105-7-363-S3.PDF]

a)

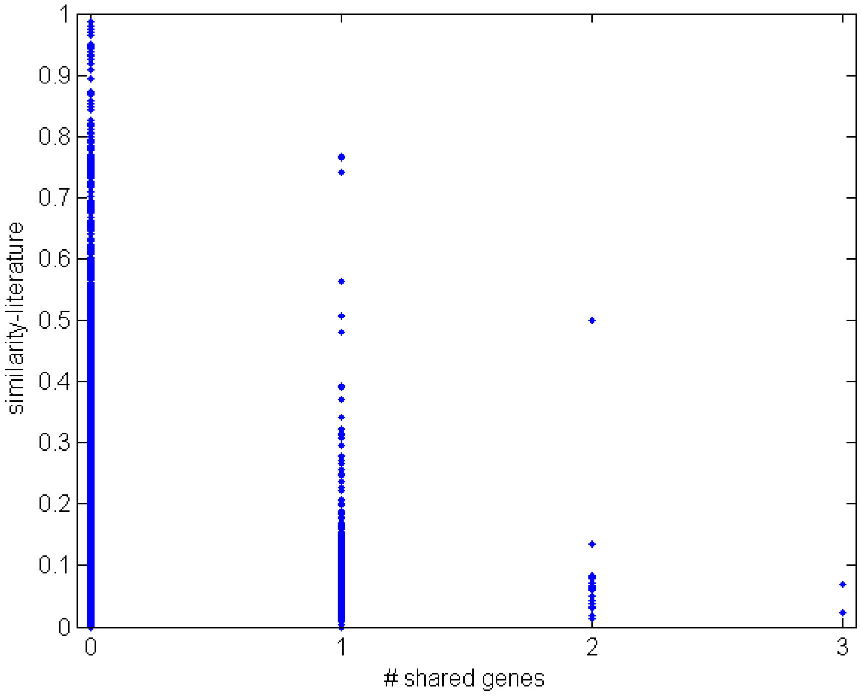

b)

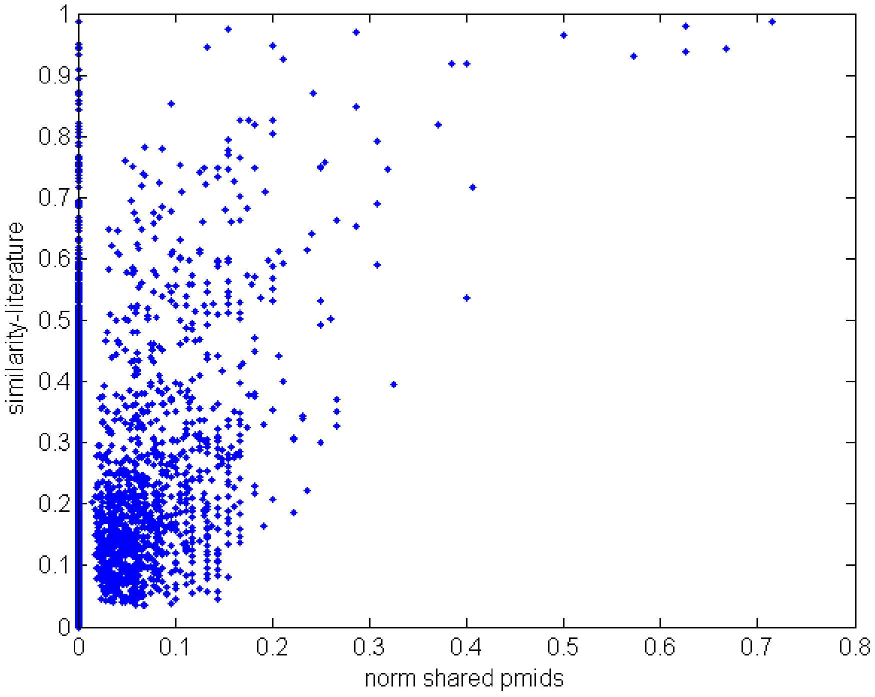

Correlation between literature similarity and shared genes/references: Scatter plots of literature-based similarity of evaluation subset against: a) the number of genes in SGD GO annotation shared by the two processes in the pair (note than no more than 3 genes are shared by any two processes); (b) the normalised number of references shared by the two biological processes.
