# Supplementary material for: A literature-based similarity metric for biological processes
Source: BMC Bioinformatics. 2006 Jul 26;7:363. doi: 10.1186/1471-2105-7-363 (PMC1579237; doi:10.1186/1471-2105-7-363)
Supplement: Additional file 4 — Correlation among similarity metrics. This file contains the correlation coefficients (using Pearson, Spearman, Kendall and uncentered dot product methods) among the four similarity metrics used for the evaluation set. [file 1471-2105-7-363-S4.PDF]

	Pearson		Spearman's rho		Kendall's tau		Uncentered	
	All	Subsume	All	Subsume	All	Subsume	All	Subsume
Slit-Slin	0.3516	0.4456	0.2143	0.5660	0.1483	0.3979	0.6321	0.8114
Slit- Czdice	0.2582	0.4401	0.1715	0.5031	0.1161	0.3579	0.7008	0.8103
Slit- Sann	0.5336	0.5794	0.2310	0.6158	0.1659	0.4365	0.5820	0.8461
Sann-Slin	0.4519	0.8461	0.1058	0.9174	0.0765	0.7602	0.5276	0.9625
Sann-Scd	0.2889	0.5860	0.1149	0.5995	0.0822	0.4267	0.3875	0.9194
Slin-Scd	0.5793	0.6631	0.6137	0.5991	0.4478	0.4257	0.7577	0.9595

Correlation among similarity metrics: correlation coefficients are calculated as: Pearson*, Spearman, Kendall and uncentered dot product. Correlations are reported for all process pairs (All), and for process pairs with inclusion relationships (Subsume). Slit (literature-based similarity); Slin (Lin similarity);Scd (Czekanowski-Dice similarity); Sann (Annotation based similarity).

*Analysis of scatter plots revealed that there is no linear relationship among the literature metric with any of the metrics used for comparison (therefore Pearson's correlation coefficient should be carefully interpreted).
