# Supplementary material for: A literature-based similarity metric for biological processes
Source: BMC Bioinformatics. 2006 Jul 26;7:363. doi: 10.1186/1471-2105-7-363 (PMC1579237; doi:10.1186/1471-2105-7-363)
Supplement: Additional file 5 — Similar biological processes according to the literature. This file contains the 49 biological process pairs which are similar according to literature and similar less than average according to both ontology-based metrics. The first 10 pairs correspond to Table 1 in the full text article. [file 1471-2105-7-363-S5.PDF]

The 49 biological process pairs which are highly similar according to literature (Slit>0.4345) and similar less than average according to both ontology-based metrics (Scd<0.4025 and Slin<0.14). CG: Number of genes shared by both processes (accounting for ontological relationships). G_A and G_B correspond to the number of genes in first and second process, respectively.

Slit	Scd	Slin	CG	G_A	G_B	Biological Process (A)	Biological Process (B)
0.96	0.23	0.03	7	7	13	phosphoinositide dephosphorylation	inositol lipid-mediated signaling
0.93	0.37	0.04	0	5	29	high affinity iron ion transport	iron ion homeostasis
0.91	0.37	0.04	0	5	7	copper ion import	copper ion homeostasis
0.81	0.29	0.05	13	17	104	Rho protein signal transduction	establishment of cell polarity (sensu Fungi)
0.75	0.21	0.04	0	7	81	cell wall chitin biosynthesis	cell budding
0.75	0.32	0.05	1	6	9	intracellular copper ion transport	cytochrome c oxidase complex assembly
0.73	0.40	0.00	1	6	287	mitochondrial signaling pathway	protein localization
0.73	0.32	0.04	0	6	7	intracellular copper ion transport	copper ion homeostasis
0.73	0.36	0.05	1	5	30	DNA replication checkpoint	DNA strand elongation
0.69	0.36	0.05	1	5	25	DNA replication checkpoint	DNA replication initiation
0.67	0.21	0.03	0	6	7	cytokinesis, completion of separation	cell wall chitin biosynthesis
0.64	0.24	0.06	5	5	3467	leucine biosynthesis	metabolism
0.62	0.30	0.04	0	9	15	fatty acid beta-oxidation	protein targeting to peroxisome
0.61	0.36	0.04	1	4	148	agglutination during conjugation with cellular fusion	cell wall organization and biogenesis
0.59	0.29	0.04	0	6	9	protein import into peroxisome matrix	fatty acid beta-oxidation
0.59	0.36	0.05	2	13	29	branched chain family amino acid biosynthesis	mitochondrial genome maintenance
0.59	0.36	0.04	3	6	29	siderophore-iron transport	iron ion homeostasis
0.59	0.21	0.00	0	7	16	copper ion homeostasis	response to metal ion
0.58	0.35	0.10	5	47	152	protein targeting to mitochondrion	protein complex assembly
0.57	0.36	0.13	0	6	127	allantoin catabolism	negative regulation of transcription
0.57	0.32	0.04	0	15	17	actin cortical patch assembly	Rho protein signal transduction
0.56	0.29	0.00	0	12	102	response to salt stress	cell ion homeostasis
0.53	0.31	0.07	2	38	68	peroxisome organization and biogenesis	positive regulation of transcription
0.53	0.38	0.05	10	17	105	Rho protein signal transduction	actin cytoskeleton organization and biogenesis
0.52	0.37	0.05	1	5	80	DNA replication checkpoint	DNA-dependent DNA replication
0.52	0.26	0.05	0	5	19	negative regulation of translation	ribosomal large subunit biogenesis
0.51	0.20	0.00	0	5	16	copper ion import	response to metal ion
0.50	0.36	0.05	0	5	12	DNA replication checkpoint	DNA unwinding during replication
0.50	0.32	0.05	0	5	12	DNA replication checkpoint	postreplication repair
0.50	0.40	0.06	4	5	559	DNA replication checkpoint	DNA metabolism
0.49	0.40	0.06	0	12	101	fatty acid biosynthesis	mitochondrion organization and biogenesis
0.49	0.37	0.04	2	11	25	regulation of cell growth	G1 phase of mitotic cell cycle
0.49	0.36	0.05	2	25	91	G1 phase of mitotic cell cycle	protein amino acid phosphorylation
0.49	0.35	0.05	6	17	63	Rho protein signal transduction	actin filament organization
0.48	0.26	0.07	13	13	3467	branched chain family amino acid biosynthesis	metabolism
0.47	0.36	0.12	0	6	68	allantoin catabolism	positive regulation of transcription
0.47	0.27	0.05	1	5	209	negative regulation of translation	ribosome biogenesis
0.47	0.33	0.06	5	13	559	DNA damage checkpoint	DNA metabolism

0.47	0.21	0.04	0	6	13	mitochondrial signaling pathway	glutamate biosynthesis
0.47	0.29	0.04	1	20	22	nuclear migration	mitotic spindle checkpoint
0.47	0.19	0.03	0	5	9	leucine biosynthesis	flocculation (sensu Saccharomyces)
0.46	0.21	0.00	2	5	183	DNA replication checkpoint	response to DNA damage stimulus
0.46	0.24	0.05	7	17	65	Rho protein signal transduction	bud site selection
0.46	0.39	0.06	1	5	103	DNA replication checkpoint	DNA replication
0.45	0.27	0.06	28	91	171	protein amino acid phosphorylation	signal transduction
0.45	0.24	0.04	1	6	17	traversing start control point of mitotic cell cycle	Ras protein signal transduction
0.44	0.37	0.07	1	19	48	ribosomal large subunit biogenesis	translational initiation
0.44	0.35	0.05	0	6	84	protein import into mitochondrial intermembrane space	aerobic respiration
